# Keratin 18 attenuates estrogen receptor α-mediated signaling by sequestering LRP16 in cytoplasm

**DOI:** 10.1186/1471-2121-10-96

**Published:** 2009-12-26

**Authors:** Yuanguang Meng, Zhiqiang Wu, Xiaoyun Yin, Yali Zhao, Meixia Chen, Yiling Si, Jie Yang, Xiaobing Fu, Weidong Han

**Affiliations:** 1 Department of Molecular Biology, Institute of Basic Medicine, Chinese PLA General Hospital, 28 Fu Xing Road, Beijing, 100853, China; 2 Department of Obstetrics and Gynecology, Chinese PLA General Hospital, 28 Fu Xing Road, Beijing, 100853, China

## Abstract

**Background:**

Oncogenesis in breast cancer is often associated with excess estrogen receptor α(ERα) activation and overexpression of its coactivators. LRP16 is both an ERα target gene and an ERα coactivator, and plays a crucial role in ERα activation and proliferation of MCF-7 breast cancer cells. However, the regulation of the functional availability of this coactivator protein is not yet clear.

**Results:**

Yeast two-hybrid screening, GST pulldown and coimmunoprecipitation (CoIP) identified the cytoplasmic intermediate filament protein keratin 18 (K18) as a novel LRP16-interacting protein. Fluorescence analysis revealed that GFP-tagged LRP16 was primarily localized in the nuclei of mock-transfected MCF-7 cells but was predominantly present in the cytoplasm of K18-transfected cells. Immunoblotting analysis demonstrated that the amount of cytoplasmic LRP16 was markedly increased in cells overexpressing K18 whereas nuclear levels were depressed. Conversely, knockdown of endogenous K18 expression in MCF-7 cells significantly decreased the cytoplasmic levels of LRP16 and increased levels in the nucleus. CoIP failed to detect any interaction between K18 and ERα, but ectopic expression of K18 in MCF-7 cells significantly blunted the association of LRP16 with ERα, attenuated ERα-activated reporter gene activity, and decreased estrogen-stimulated target gene expression by inhibiting ERα recruitment to DNA. Furthermore, BrdU incorporation assays revealed that K18 overexpression blunted the estrogen-stimulated increase of S-phase entry of MCF-7 cells. By contrast, knockdown of K18 in MCF-7 cells significantly increased ERα-mediated signaling and promoted cell cycle progression.

**Conclusions:**

K18 can effectively associate with and sequester LRP16 in the cytoplasm, thus attenuating the final output of ERα-mediated signaling and estrogen-stimulated cell cycle progression of MCF-7 breast cancer cells. Loss of K18 increases the functional availability of LRP16 to ERα and promotes the proliferation of ERα-positive breast tumor cells. K18 plays an important functional role in regulating the ERα signaling pathway.

## Background

Estrogen receptor α (ERα), a member of the nuclear receptor (NR) superfamily of transcription factors, plays a crucial role in the control of epithelial cell proliferation and mammary gland development [1,2] as well as in the development and progression of breast cancer [3,4]. Classically, ERα is activated by estrogen binding, and this leads to receptor phosphorylation, dimerization, and to recruitment of coactivators to the estrogen-bound receptor complex [5]. Oncogenesis in breast cancer frequently involves excessive activation of the ERα signaling due primarily to overexpression of ERα and/or its coactivators [6-9]. Factors that affect the balance of ERα and its cofactors in breast cancer cells can modulate ERα signaling and thereby alter the cell growth response to estrogen stimulation. Human MCF-7 breast cancer cells express functional ERα and display estrogen-dependent growth, and have been widely used as an in vitro model for studying the regulatory mechanisms of ERα action in estrogen-dependent breast cancer [10,11].

Most coactivator proteins contain different activation domains or enzyme activity modules that include classical histone acetylase, bromo, chromo, Su(var) 3-9, Enhancer of zeste, Trithorax and ATPase domains, by which coactivators facilitate the assembly of the transcription initiation complex through their chromatin remodeling activities [12,13]. LRP16 is a member of the macro domain superfamily with a simple structure compared to other members because it contains only a single stand-alone macro module in its C-terminal region [14,15]. LRP16 was previously identified as a target gene for both ERα and the androgen receptor (AR) [15,16]. The proximal region (nt -676 to -24) of the human LRP16 promoter contains a 1/2 ERE/Sp1 site and multiple GC-rich elements that confer estrogen responsiveness and is sufficient for estrogen action [17,18]. LRP16 protein interacts with both ERα and AR and enhances their transcriptional activities in a ligand-dependent manner, thus establishing a positive feedback regulatory loop between LRP16 and ERα/AR signal transduction [15,19]. In addition, LRP16 has also been reported to act as a potential coactivator that amplifies the transactivation of 4 other NRs [15]. Overexpression of LRP16 can stimulate the proliferation of MCF-7 breast cancer cells by enhancing estrogen-stimulated transcription mediated by ERα [16,19]. Inhibition of *LRP16 *gene expression significantly suppresses the proliferative activity and invasiveness of estrogen-responsive epithelial cancer cells [19,20]. Consistent with findings in cell culture, a positive correlation was found between LRP16 mRNA levels and the progression of primary breast cancers [21]. Although the mechanisms of estrogen regulation of LRP16 expression and the functional role of LRP16 in ERα-mediated transcriptional regulation are relatively well characterized, the regulation of the functional availability of this coactivator protein is unclear.

The cytoskeleton of epithelial cells is predominantly formed by intermediate filament protein keratins (KRTs) that are subclassified into type I (acidic, KRT9 through KRT20) and type II (neutral-basic, KRT1-KRT8) families [22]. K18 (KRT18) is expressed in single-layer epithelial cells of the human body and is localized in the cytoplasm and perinuclear region. In the normal mammary epithelium, K18 is expressed in the luminal cells that represent the differentiation compartment [23]. K18 has been recognized for many years as an epithelial marker in diagnostic histopathology [24]. The level of K18 expression has been inversely associated with the progression of breast cancer: 25% to 80% of all breast carcinomas exhibit loss of K18 expression and this is associated with significantly poorer prognosis [25-30]. Transfection of K18 into ERα-negative MDA-MB-231 breast cancer cells caused significant reduction of malignancy both *in vitro *and *in vivo *[31]. Results from cell-culture experiments and clinicopathological parameter analyses have also revealed a relationship between decreased amounts of K18 in the cytoplasm and increased proliferative activity of breast cancer cells [27,28]. These previous studies suggest that K18 plays an important role in tumor progression in breast cancer patients, but the molecular mechanisms are poorly understood.

In the present study we first used the yeast two-hybrid system to investigate proteins interacting with LRP16. This revealed that K18 physically interacts with LRP16 through its C-terminal region. Moreover, K18 binding sequesters LRP16 in the cytoplasm and prevents its enhancement of ERα-mediated transcription in MCF-7 cells. Using estrogen-responsive MCF-7 cells as a model we have demonstrated that K18 modulates both estrogen activation of ERα target genes and cell cycle progression. These results suggest that loss of K18 expression in ERα-positive breast cells, and failure of cytoplasmic sequestration of the ERα coactivator LRP16, may contribute to tumor proliferation by increasing ERα signaling in the nucleus.

## Results

### K18 is a novel interactor of LRP16

**Figure 1:**
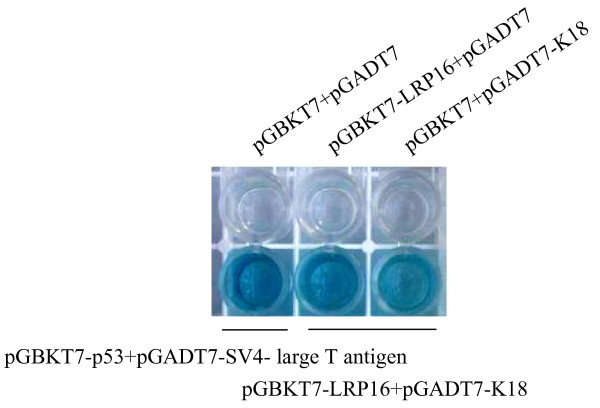
**K18 interacts with LRP16 in yeast cells**. Yeast AH109 cells were transformed with the indicated GAL4-DBD (DNA Binding Domain) and GAL4-AD (Activation Domain) chimeric constructs and β-galactosidase activity was measured by a liquid *o*-nitrophenyl-β-D-galactoside (ONPG) assay. The experiment was repeated 3 times, and 2 different yeast transformants were used for each measurement. The interaction of p53 with SV40 large T-antigen protein provided a positive control.

The yeast two-hybrid system was used to screen for new polypeptides interacting with LRP16. Sequences from a MCF-7 breast cancer cell cDNA library were screened for binding to LRP16; this identified nine clones corresponding to 12 different potential LRP16-binding proteins. One such cDNA clone was found to contain a full-length coding sequence (amino acids 1-430) for the cytokeratin K18. The specificity of the interaction between LRP16 and K18 was demonstrated by chromogenic assay using X-Gal; no staining developed using either factor alone or in pairwise controls containing only the Gal4 activation domain (AD) or the Gal4 DNA binding domain (DBD) (Figure 1).

**Figure 2:**
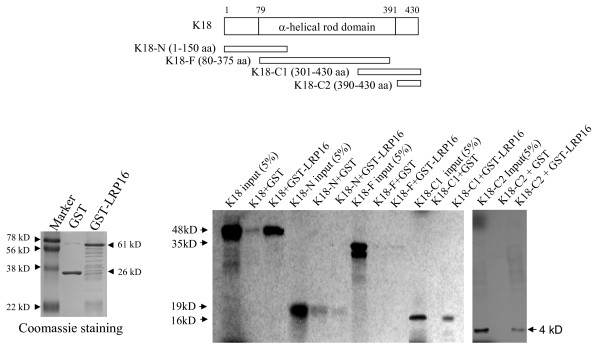
**K18 interacts with LRP16 protein by its C-terminal region mediation**. Top panel, schematic illustration of K18 and its mutants. GST-pulldown assays were performed with *in vitro*-translated [^35^S]-labeled K18 and its mutants in the presence of GST-LRP16 fusion protein (bottom panel). GST protein was used as a control. K18-C1 was run on a 21% SDS-PAGE gel; the others were run on 12% gels.

**Figure 3:**
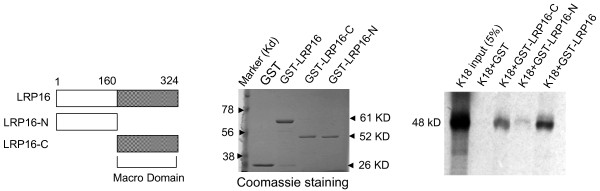
**LRP16 interacts with K18 by its macro domain mediation**. Left panel schematic illustration of LRP16 and its mutants. Middle panel, Coomassie blue-stained GST, GST-LRP16-N and GST-LRP16-C. Right panel, GST alone, GST-LRP16 mutants or GST-LRP16 were used to pull down full-length K18.

To confirm the specificity of the interaction between K18 and LRP16 we analyzed glutathione S-transferase (GST) fusion proteins and *in vitro*-translated proteins by pulldown assays. GST-LRP16 efficiently bound to *in vitro*-translated ^35^S-labeled full-length K18 (Figure 2). A series of K18 deletion constructs were then used in GST pull-down assays to identify the region within K18 that is required for LRP16 binding. GST-LRP16 failed to bind to either K18-N (amino acids 1-150) or K18-F (80-375) but bound strongly to both K18-C1 (301-430) and K18-C2 (390-430) (Figure 2). We then tested N- and C-terminal LRP16 deletion constructs for K18 binding. Full-length K18 polypeptide bound strongly to GST-LRP16-C (amino acids 161-324) but only weakly to GST-LRP16-N (1-160); K18 failed to bind to GST alone (Figure 3). Together these results indicate that the interaction between K18 and LRP16 is mediated primarily by the C-terminal region of K18 and the single macro domain of LRP16.

**Figure 4:**
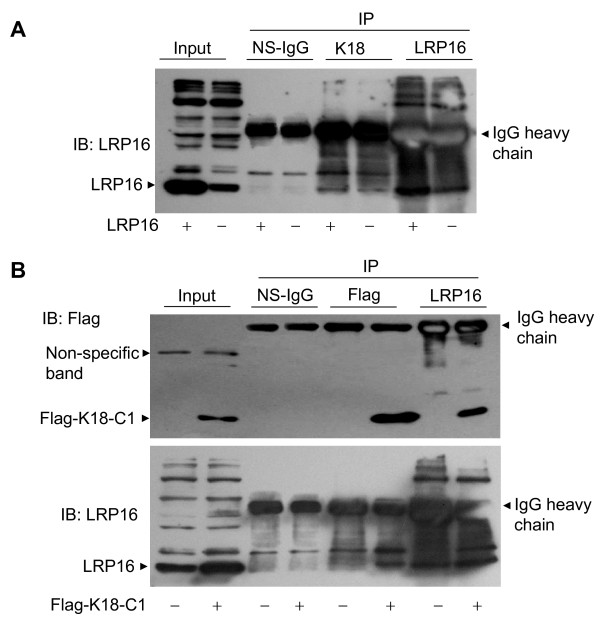
**K18 interacts with LRP16 protein *in vivo***. **A**, MCF-7 cells were transfected with the LRP16 expression vector or empty vector. Cell lysates were immunoprecipitated (IP) and immunoblotted (IB) with the indicated antibodies. **B**, MCF-7 cells were transfected with Flag-tagged K18-C1 (301-430 aa) expression vector or the corresponding empty vector. Cell lysates were immunoprecipitated and immunoblotted with the indicated antibodies.

We then used co-immunoprecipitation (CoIP) to confirm that K18 interacts with LRP16 in mammalian cells. A pcDNA3.1 expression vector directing the expression of LRP16 (pcDNA3.1-LRP16) was transfected into MCF-7 cells; cell lysates were then immunoprecipitated with antibodies directed against either K18 or LRP16. Precipitates were resolved by gel electrophoresis and probed with antibody against LRP16. The empty pcDNA3.1 expression vector provided a negative control. An intense band corresponding to LRP16 was detected in anti-K18 antibody immunoprecipitates from LRP16-overexpressing MCF-7 cells (Figure 4A, lane 5). In addition, a weak band corresponding to endogenous LRP16 was detected in anti-K18 immunoprecipitates from vector-transfected MCF-7 cells (Figure 4A, lane 6). Nonspecific IgG antibody failed to immunoprecipitate LRP16 (lanes 3 and 4 in Figure 4A). To confirm the specificity of LRP16-K18 complex formation we transiently transfected Flag-tagged empty vector or Flag-K18-C1 (amino acids 301-430) into MCF-7 cells for CoIP assays. As shown in Figure 4B, the exogenous Flag-K18-C1 and the endogenous LRP16 could be reciprocally coimmunoprecipitated by use of anti-Flag and/or anti-LRP16 antibodies. These results confirm that K18 can bind to LRP16 in MCF-7 breast cancer cells.

### K18 modulates the nucleo-cytoplasmic localization of LRP16 in MCF-7 cells

K18, a member of the family of intermediate filament keratins, is localized to the cytoplasm and is not generally found in the nucleus. By contrast, LRP16 acts as a common coactivator for the nuclear receptors ERα and AR, and this implies that LRP16 is present in the nucleus. The physical association between K18 and LRP16 therefore suggested the possibility that K18 might modulate the nucleo-cytoplasmic distribution of LRP16.

**Figure 5:**
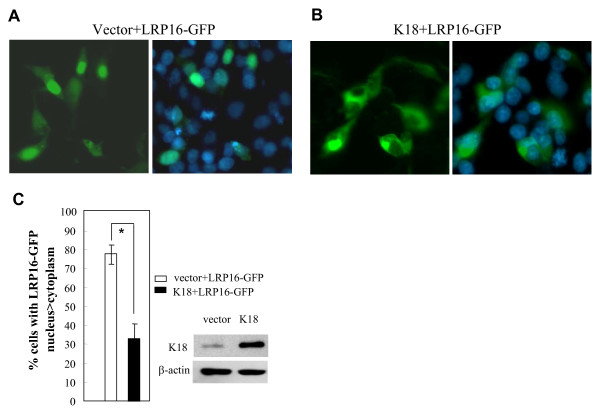
**K18 sequesters LRP16-GFP fusion protein in the cytoplasm from nucleus**. **A, B, C**, The LRP16-GFP expression vector was transfected or cotransfected with K18 into MCF-7 cells and the proportion of cells displaying LRP16-GFP in the nucleus was determined. All experiments were performed in triplicate and were repeated at least 3 times; the results are expressed as mean ± SEM. A and B show representative fluorescence patterns.

To address this possibility we examined whether increased K18 expression in MCF-7 cells might alter the subcellular distribution of a LRP16-GFP fusion protein. As expected for a nuclear protein, LRP16-GFP fluorescence was found primarily in the nucleus, and nuclear fluorescence was detected in 78% of GFP-positive cells; cytoplasmic fluorescence was only detected in 22% of GFP-positive cells cotransfected with empty vector (Figure 5A and 5C). However, the distribution was reversed when cells expressing LRP16-GFP were cotransfected with a construct directing the expression of K18. Here nuclear fluorescence was detected in only 32% of GFP-positive cells whereas 68% exhibited cytoplasmic localization (Figure 5B and 5C). These results suggest that the ectopic expression of K18 can sequester LRP16 into cytoplasm.

**Figure 6:**
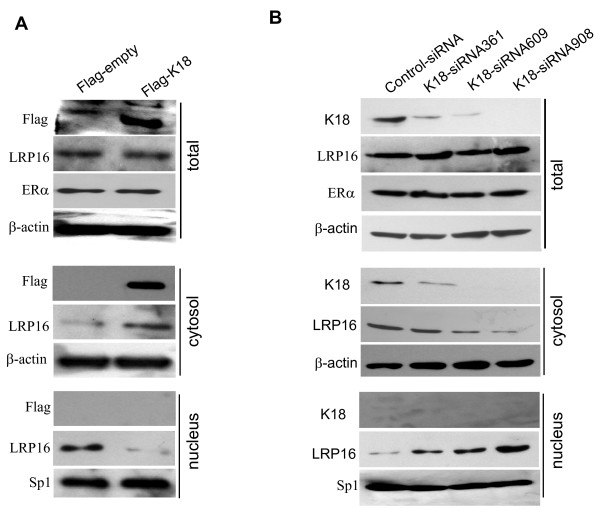
**Differential expression of K18 regulates the nucleo-cytoplasmic distribution of the endogenous LRP16 protein in MCF-7 cells**. **A**, MCF-7 cells were transiently transfected with Flag-tagged K18. Total, nuclear and cytoplasmic proteins were extracted 48 h after transfection and were subjected to immunoblotting analysis with the indicated antibodies. **B**, MCF-7 cells were transiently transfected with K18-specific siRNAs or the control siRNA. Total, nuclear and cytoplasmic proteins were extracted 48 h after transfection and were subjected to immunoblotting analysis with the indicated antibodies. β-actin was used as a loading control for total protein extracts and cytoplasmic extracts. Transcription factor Sp1 expressed constitutively in the nucleus was used as a loading control for nuclear protein extracts.

To further confirm this finding transfected cells were physically separated into cytoplasmic and nuclear fractions and the distribution of LRP16 was analyzed by immunoblotting. MCF-7 cells were transfected with a K18 expression construct, Flag-K18, or with empty vector, and total, cytoplasmic and nuclear extracts were analyzed using antibody to LRP16. As shown in Figure 6A, total LRP16 protein levels were not altered by ectopic expression of K18 in MCF-7 cells; by contrast, K18 expression significantly increased LRP16 levels in the cytoplasm and reduced the proportion present in the nucleus, a finding consistent with K18 sequestration of LRP16 in the cytoplasm.

To address whether endogenous K18 polypeptide also sequesters LRP16 in the cytoplasm we studied the effects of inhibiting endogenous K18 expression on the distribution of LRP16. Three different small interfering RNA (siRNA) duplexes directed against human K18 mRNA, siRNA361, 609 and 908, were designed and transfected into MCF-7 cells; levels of LRP16 in the total, nuclear, and cytoplasmic fractions were measured by immunoblotting as before. Levels of K18 polypeptide were significantly reduced by transfection with all three K18 siRNAs as compared to cells transfected with a control siRNA; knock-down activity declined in the order siRNA361, 609, 908 (Figure 6B). None of the siRNAs affected the total levels of LRP16, but knockdown of endogenous K18 expression with the three siRNAs led to a significant and graded decrease in cytoplasmic LRP16 levels and a corresponding graded increase in nuclear levels (Figure 6B). Similar effects of K18 overexpression and knockdown on the subcellular distribution of endogenous LRP16 protein were also observed in human cervical cancer HeLa cells (data not shown). Together these data indicate that endogenous K18 sequesters LRP16 in the cytoplasm.

### K18 binding to LRP16 modulates ERα signaling

**Figure 7:**
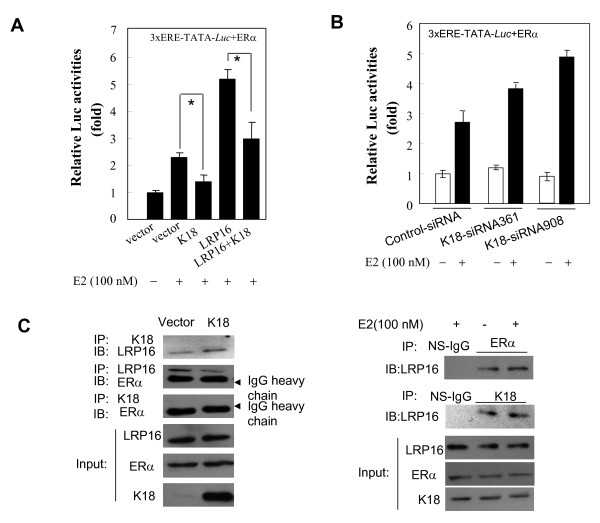
**K18 modulates E2-activated reporter gene activity and the binding of LRP16 to ERα in MCF-7 cells**. **A**, MCF-7 cells were grown in phenol-red free media stripped of steroids for at least 3 days, then cotransfected with 3×ERE-TATA-*Luc *reporter, ERα expression vector and the indicated vectors. 36 h after transfection, cells were treated with E2 (100 nM) or dimethyl sulphoxide (DMSO) for 6 h before luciferase assay. The relative luciferase activity levels were normalized by use of mock effector transfection and arbitrarily assigned a value of 1. All experiments were performed in triplicate and were repeated at least 3 times; results are expressed as means ± SEM. *, *P *< 0.05. **B**, MCF-7 cells were grown in phenol red-free media stripped of steroids for at least 3 days, then cotransfected with the indicated siRNA oligonucleotides, 3× ERE-TATA-*Luc *reporter and the ERα expression vector. 42 h after transfection, cells were treated with E2 (100 nM) or vehicle (DMSO) for 6 h before luciferase assay. Relative luciferase activity levels were normalized to transfections with control siRNA and were arbitrarily assigned a value of 1. All experiments were performed in triplicate and were repeated at least 3 times; results are expressed as means ± SEM. **C**, MCF-7 cells were transiently transfected with K18 expression vector or the corresponding empty vector. Cell lysates were immunoprecipitated and immunoblotted with the indicated antibodies (left panel). MCF-7 cells were cultured in phenol-red free media stripped of steroids for at least 3 days, then treated with E2 (100 nM) for 1 h and subjected to CoIP analysis by the use of the indicated antibodies (right panel). Ns-IgG, non-specific IgG.

Our previous studies demonstrated that LRP16 is a coactivator of ERα in the nucleus and that knockdown of LRP16 in MCF-7 cells can significantly attenuate estradiol (E2)-stimulated ERα signaling [19]. Because K18 can sequester LRP16 from the nucleus into the cytoplasm it is possible that K18 expression might modulate ERα signaling. To explore this possibility we assayed E2-activation of a construct in which expression of a luciferase gene (*Luc*) is under the control of three estrogen-response elements (EREs). Cotransfection of MCF-7 with the 3× ERE-TATA-*Luc *reporter construct and with ERα and the pcDNA3.1 empty vector revealed background luciferase activity (Figure 7A, lane 1). Reporter gene activity increased by 2.3-fold on treatment with E2 (100 nM) (Figure 7A, lane 2). However, activation was significantly attenuated by cotransfection with a construct directing K18 expression (Figure 7A, lane 3). Consistent with our previous report [19], the E2-activated reporter system was further augmented by LRP16 transfection (Figure 7A, lane 4), but this LRP16-enhanced reporter gene activity was also markedly impaired by cotransfection with the K18 expression construct (Figure 7A, lane 5). Comparison of reporter gene activities in lanes 3 and 5 revealed that K18 suppression of E2-stimulated ERα transcriptional activity was efficiently antagonized by overexpression of LRP16. We next used RNA interference in the cotransfection system to explore K18 suppression of reporter gene expression in MCF-7 cells. siRNA directed against K18 was found to enhance ERα-mediated transactivation in the presence of E2. In the absence of E2, however, knockdown of endogenous K18 failed to increase reporter gene expression (Figure 7B). Furthermore, CoIP analysis revealed that ectopic K18 expression in MCF-7 cells markedly attenuated the association of ERα with LRP16; there was no evidence for any direct interaction between K18 and ERα (Figure 7C, left panel). Consistent with our previous observations [19], E2 stimulation enhanced the interaction between LRP16 and ERα but had no effect on the interaction between K18 and LRP16 (Figure 7C, right panel). Together these results indicate that K18 can suppress E2-stimulated ERα transactivation by blunting the binding of LRP16 to ERα.

**Figure 8:**
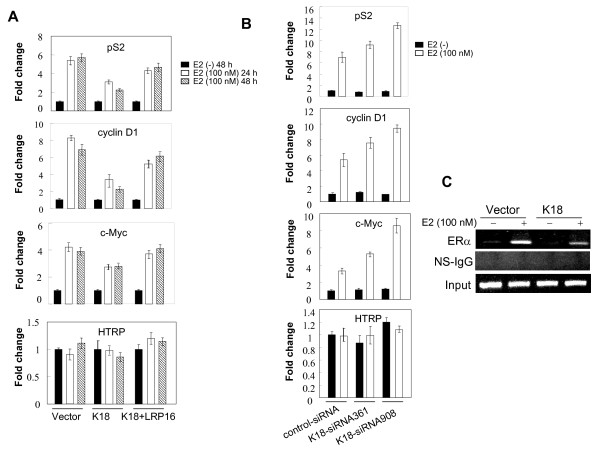
**K18 modulates E2-stimulated expression of ERα target genes and the recruitment of ERα to its target DNA in MCF-7 cells**. **A**, MCF-7 cells were grown in phenol-red free media stripped of steroids for at least 3 days, then cotransfected with the indicated vectors and cultured for the indicated times. Before total RNA was extracted, the cells were treated with E2 (100 nM) or vehicle (DMSO) for 1 h. Expression of the indicated transcript abundance was analyzed by quantitative RT-PCR (qPCR). *HPRT *was used as the internal control. All experiments were repeated at least 3 times; results are expressed as means ± SEM. **B**, MCF-7 cells were grown in phenol-red free media stripped of steroids for at least 3 days, then cotransfected with the indicated siRNAs. After 47 h, cells were treated with E2 (100 nM) or vehicle (DMSO) for 1 h and were subjected to qPCR analysis. Transcript abundance was analyzed by qPCR. *HPRT *was used as the internal control. All experiments were repeated at least 3 times; results are expressed as means ± SEM. **C**, MCF-7 cells, grown in phenol-red free media stripped of steroids, were transiently transfected with K18 expression vector or empty vector. 40 h post-transfection, cells were treated with E2 (100 nM) for 1 h and were subjected to ChIP analyses with the indicated antibodies.

To address whether K18 affects E2 induction of ERα target genes in MCF-7 cells we used quantitative PCR to measure mRNA expression levels of the *pS2*, *cyclin D1*, and *c-Myc *genes whose expression is known to be E2-regulated in MCF-7 cells [19]. As shown in Figure 8A, E2 treatment produced a marked increase in the mRNA levels of *pS2*, *cyclin D1*, and *c-Myc *but not of the control gene *HPRT*. However, this induction was attenuated by overexpression of K18. Overexpression of LRP16 efficiently relieved K18 inhibition of E2-induced expression of these target genes. We next analyzed E2 induction of these target genes in MCF-7 cells transfected with K18 siRNAs. As shown in Figure 8B, knockdown of endogenous K18 expression greatly increased the level of E2-induced up-regulation of *pS2*, *cyclin D1*, and *c-Myc *mRNA.

To confirm that the effects of K18 are mediated at the transcriptional level we used chromatin immunoprecipitation (ChIP) assays to analyze ERα recruitment at the *pS2 *promoter region. As shown in Figure 8C, ERα binding at the *pS2 *promoter was significantly increased in the presence of E2, but binding was substantially blunted by overexpression of K18.

**Figure 9:**
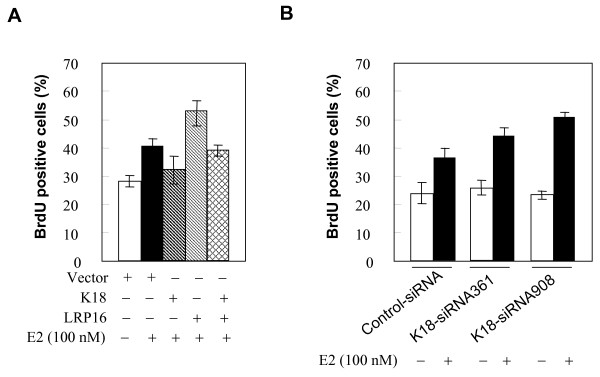
**K18 modulates E2-promoted cell cycle progression of MCF-7 cells**. **A**, MCF-7 cells were grown in phenol-red free media stripped of steroids for at least 3 days, then transiently cotransfected with the indicated vectors. The pEGFP-N1 plasmid was included to identify transfected cells. After 36 h, cells were treated with or without E2 (100 nM) for an additional 12 h, then labeled with BrdU (10 μM) for 2 h and immunostained for BrdU with a Cy3-conjugated secondary antibody. Cells were assessed for GFP and BrdU, and the proportion of transfected cells positive for BrdU was scored. **B**, MCF-7 cells were grown in phenol-red free media stripped of steroids for at least 3 days, then transiently cotransfected with the indicated siRNA duplexes. After 36 h, cells were treated with or without E2 (100 nM) for an additional 12 h and assessed for BrdU incorporation as in A. All experiments in A and B were performed in triplicate and were repeated at least 3 times; results are expressed as means ± SEM.

We previously reported that knockdown of LRP16 can markedly inhibit E2-stimulated growth of MCF-7 cells [19]. To determine whether the K18-LRP16 association might modulate the E2-stimulated transition from the G1 to S phase of the cell cycle, MCF-7 cells were transfected with constructs directing the expression of K18 and/or LRP16 as well as with a GFP expression plasmid. The extent of DNA synthesis was assessed by incorporation of BrdU into GFP-positive cells. As shown in Figure 9A (lane 1), S-phase entry was 13% greater in E2-treated cells than in control cells (lane 2), whereas in cells transfected with a construct expressing K18 there was only a 4% increase in S-phase entry in K18-transfected cells (lane 3). Furthermore, overexpression of LRP16 substantially increased E2-stimulated S-phase entry (lane 4); however, this increase was blocked by K18 overexpression (lane 5). We next performed BrdU incorporation assays on MCF-7 cells transfected with K18 siRNA. As shown in Figure 9B, transfection of K18-specific siRNAs greatly increased E2-promoted S-phase entry compared to controls. Together these data indicate that, by sequestering LRP16 in the cytoplasm, K18 can effectively inhibit estrogen-promoted cell-cycle progression of estrogen-sensitive MCF-7 breast cancer cells.

## Discussion

Regulation of transcription factor and cofactor activity by subcellular compartmentalization is well documented [32-34]. A common mechanism is sequestration of the factor into inactive compartments, and this typically takes place via direct or indirect association with the cytoskeleton [35-38]. LRP16 is a new type of ERα coactivator that augments the receptor's transcriptional activity in a ligand-dependent manner and can have a profound impact on the final output of cellular signaling [19]. LRP16 is though to modulate ERα activity in the nucleus; in the present paper we have confirmed that a LRP16-GFP fusion protein localizes primarily to MCF-7 cell nuclei. We also report a new LRP16 ligand, K18, identified by yeast two-hybrid screening. K18 is a member of the family of intermediate filament proteins that contribute to cytoskeletal architecture. In the present study we report that K18 binds to and sequesters LRP16 in the cytoplasm, thus preventing its nuclear action and attenuating both E2-induction of ERα target genes and E2-stimulated cell cycle progression of MCF-7 cells. These findings underscore the functional role of K18 in regulating the ERα signaling pathway.

LRP16, a member of the macro domain protein superfamily, contains a single stand-alone macro module in its C-terminal region [14,15]. We recently demonstrated that LRP16 is a non-redundant coactivator of both ERα and AR [15,19]. LRP16 was also able to interact with another 4 nuclear receptors (NRs) *in vitro*, including estrogen receptor β(ERβ), the glucocorticoid receptor, and peroxisome proliferator-activated receptors α and γ, and can efficiently amplify the transactivation of these NRs in a ligand-dependent manner [15]. Our finding that K18 binds to and sequesters LRP16 in the cytoplasm suggests that differential tissue expression of K18 could constitute a new layer in the regulatory cascade of signaling pathways in which LRP16 participates.

Keratins (KRTs) provide mechanical stability to tissues, as evidenced by the range of pathological phenotypes seen in patients bearing mutations in epidermal keratins [39]. The intermediate filament network in simple glandular epithelial cells predominantly consists of heterotypic complexes of KRTs K8 and K18. Additional evidence for a more widespread role of KRTs comes from mouse gene knockout studies. Double deletion of the genes encoding K18 and K19 results in complete loss of a functional cytokeratin skeleton and embryonic lethality [40]. The assembly of intermediate filament involves several steps during which the α-helical rod domain of the cytokeratin molecules plays a central role [41-43]. The head and the tail domains are not thought to be part of the filamentous backbone, and instead these protrude laterally and contribute to profilament and intermediate filament packing and to intermediate filament interaction with other cellular components [44-46]. By associating with signal transduction factors K18 may modulate both intracellular signaling and gene transcription. For example, K18 is known to bind specifically to the tumor necrosis factor (TNF) receptor type 1(TNFR1)-associated death domain protein (TRADD) through its N-terminal region and prevent TRADD from binding to activated TNFR1, thus attenuating TNF-induced apoptosis in simple epithelial cells [44].

We report here that K18 binding to LRP16 is primarily mediated by the C-terminal region of K18 and the single macro domain of LRP16. We used two independent approaches, including subcellular localization analysis of GFP-tagged LRP16 and cytoplasmic/nuclear LRP16 protein expression analyses, to demonstrate that ectopic K18 expression in MCF-7 cells sequesters LRP16 in the cytoplasm. Conversely, knockdown of *K18 *gene expression increased the nuclear localization of LRP16. By binding to and sequestering LRP16 in the cytoplasm, K18 prevents the nuclear action of LRP16 and attenuates ERα signaling, thus blunting estrogen-stimulated cell-cycle progression of ERα-positive breast cancer cells.

Accumulating evidence from clinicopathological observations has shown that the level of *K18 *gene expression correlates inversely with the progression of breast cancer [25-31,47]. Several reports have proposed that downregulation of K18 might increase the invasiveness of breast cancer cells [25-30,47]. It was previously demonstrated that overexpression of K18 in the ERα-negative and highly invasive MDA-MB-231 breast cancer cell line caused a marked reduction in the aggressiveness of the cells *in vitro *and *in vivo *but had no significant effect on cell growth rate. This change was accompanied by complete loss of the previously strong vimentin expression in the parent cell line and upregulation of adhesion proteins such as E-cadherin [31]. However, experimental studies and clinicopathological observations also revealed a significant association between K18 expression and the proliferation rate of breast cancer cells. Analysis of the association between K18 expression and different clinicopathological risk factors revealed that K18 expression is highly and significantly correlated with size (pT1-3), differentiation grade, and mitotic index of the primary tumor [27]. These parameters are a function of the proliferation rate of the primary tumor, and this suggests that there is a relationship between downregulation of K18 expression and increased proliferative activity. In addition, the expression of the proliferation-associated antigen Ki-67 is significantly associated with the downregulation of K18 in a subset of primary breast carcinomas [27]. Moreover, cell culture experiments on bone-marrow micrometastases of breast cancer have indicated that most proliferating tumor cells lack detectable expression of K18 protein [28]. These previous data suggested that K18 might make an important contribution to tumor metastasis as well as to tumor cell growth. In the present study we have demonstrated that, by blunting estrogen-stimulated ERα signaling activity, K18 can significantly suppress the growth response of MCF-7 cells to estrogen. We propose that the regulatory mechanism of ERα transactivation by the K18-LRP16 association might explain in part the relationship between K18 downregulation and increased proliferative activity of breast cancers. However, K18 loss is also associated with the metastasis of ERα-negative breast cancers (47), and it therefore appears likely that K18 can modulate breast cancer progression by more than one mechanism.

Oncogenesis in breast cancer commonly involves excess activation of ERα signaling. We previously reported that LRP16 mRNA is overexpressed in nearly 40% of all primary breast cancer samples [21]. LRP16 overexpression in breast cancer cells is tightly linked with cell proliferation and enhanced ERα activation [16,19,21]. As a functional suppressor of LRP16, K18 is frequently absent from different types of breast carcinoma [25-30]. Excess activation of ERα function in tumor cells is commonly mediated by overexpression of ERα and/or its coactivators including LRP16 [6-9,21]. We now propose a further level of regulation that can modulate ERα function in breast cancer. Loss of K18 from ERα-positive breast tumor cells releases the functional activity of LRP16, and is thus likely to promote tumor cell proliferation. Tests that evaluate the subcellular localization of LRP16 in ERα-positive breast tumor cells therefore have potential in the categorization of different clinopathological stages.

## Conclusions

In summary, these findings provide evidence that K18 binding to LRP16 leads to cytoplasmic sequestration of LRP16. By determining the nuclear availability of the receptor coactivator LRP16, K18 can not only modulate the transcriptional activity of ERα in response to estrogen but can also govern estrogen-stimulated cell cycle progression of MCF-7 cells. Loss of K18 from ERα-positive breast tumor cells releases the functional activity of LRP16, and such loss is thus likely to promote tumor proliferation. These findings underscore a functional role for K18 in regulating the ERα signaling pathway.

## Methods

### Chemicals, cell lines and small interfering RNA (siRNA)

17β-estradiol (E2) was purchased from Sigma (St Louis, MO, USA). Steroid-deprived serum was prepared as described previously [18]. Phenol-red free Dulbecco's modified Eagle's medium (DMEM) was from the Institute of Basic Medicine, Beijing Union Hospital (Beijing). MCF-7 cells were originally obtained from the American Type Culture Collection (ATCC, Rockville, MD, USA) and cultured according to ATCC instructions. Duplexes of K18 specific siRNAs 361 (sense strand, 5'-GACCATGCAAAGCCTGAAC-3'), 609 (sense strand, 5'-GAGTCAAGTATGAGACAGA-3') and 908 (sense strand, 5'-GAGGAGCTAGACAAGTACT-3') were chemically synthesized by Shanghai GeneChem Co. (Shanghai). The unrelated siRNA sequence (sense strand, 5'-GACGAACGTGTCACGTATC-3') was used as a control.

### Plasmids

The pcDNA3.1-LRP16 and the pcDNA3-Flag plasmid were described previously [15]. The human ERα expression vector pSG5-hERα was kindly provided by Dr. Hajime Nawata (Kyushu University, Japan). The reporter 3× ERE-TATA-*Luc *was provided by Prof. Donald P. McDonnell (Duke University Medical Center, Durham, NC, USA). The LRP16-GFP fusion expression vector was constructed by inserting the full-length LRP16 cDNA at the *Kpn*I and *BamH*I sites of the pEGFP-N1 vector. The yeast expression plasmid pGBKT7-LRP16 (Gal4 BD:bait gene fusion) was generated by inserting the full-length LRP16 cDNA in-frame at the *Eco*RI site of pGBKT7. To generate the GST-LRP16 fusion plasmid and its mutants GST-LRP16-N (1-160) and GST-LRP16-C (161-324) the corresponding fragments were PCR-amplified and inserted at the *Eco*RI/*Hin*dIII sites of plasmid pGEX-6p-1 (Amersham Biosciences, Freiburg, Germany). The full-length coding region of human *K18 *was amplified from GAL4 AD:K18 (pGADT7-K18) and then cloned at the *BamH*I/*EcoR*I sites of pcDNA3.1. To generate K18 deletion mutants K18-N (amino acids 1-150), K18-F (80-375), K18-C1 (301-430) and K18-C2 (390-430), the corresponding fragments were PCR-amplified and inserted at the *Eco*RI/*Xho*I sites of the pcDNA3.1 or pcDNA3-Flag vectors.

### Generation of the cDNA library and yeast two-hybrid screening

Total RNA from MCF-7 cells was extracted using TRIzol reagent (Invitrogen, Carlsbad, CA, USA) and a cDNA library was generated using the BD SMART™ kit (Clontech, Palo Alto, CA, USA) according to the manufacturer's instructions. Yeast two-hybrid screening for the identification of LRP16-interacting proteins involved the MATCHMARKER two-hybrid system 3 kit (Clontech) according to the manufacturer's instructions.

### GST pull-down assay

GST and GST fusion proteins were prepared as described previously [15]. ^35^S-labeled proteins were produced with use of a TNT-coupled *in vitro *transcription and translation system (Promega Corporation, Madison, WI, USA) with the expression vector K18 and its derivatives in pcDNA3.1.

### Extraction of cytoplasmic/nuclear proteins, co-immunoprecipitation (CoIP) and immunoblotting

Cells were cultured in 10 cm dishes and transfected with expression vectors or siRNA duplexes. 48 h after transfection, cells were harvested and lysed for CoIP or immunoblotting assays. Extraction of total, cytoplasmic, or nuclear proteins employed the ReadyPrep™ protein extraction kit (Bio-Rad Laboratories, Hercules, CA, USA) according to the instruction manual supplied by the manufacturer. For CoIP assays, cells were lysed in 500 μl lysis buffer (20 mM Tris [pH 7.4], 50 mM NaCl, 1 mM EDTA, 0.5% NP-40, 0.5% SDS, 0.5% deoxycholate, and protease inhibitors). To efficiently solubilize keratins, cells were treated with 2% Empigen BB (Sigma) as described previously [48]. Lysate aliquots (500 μg; 1 μg/μl) were precleared with 50 μl of protein A-Sepharose beads (Upstate Biotechnology, Lake Placid, NY, USA) for 2 h at 4°C. Appropriate amounts of rabbit anti-LRP16, rabbit anti-Flag (Sigma), rabbit anti-ERα(Santa Cruz Biotechnology, Santa Cruz, CA, USA) or rabbit nonspecific IgG (Clontech) was then added and incubated overnight at 4°C. Preblocked agarose beads (100 μl) were then added to the antibody/lysate mixture and incubation was continued for a further 2 h at 4°C. After washing (3×), bound proteins were eluted in SDS sample buffer, resolved by SDS-PAGE, and analyzed by immunoblotting. The rabbit and mouse anti-LRP16 antibodies were as described previously [15]. Antibodies used for immunoblotting were mouse anti-K18 (Abgent, San Diego, CA, USA), mouse monoclonal anti-Flag (Sigma), rabbit anti-ERα, mouse anti-Sp1 and rabbit anti-β-actin (Santa Cruz Biotechnology).

### Quantitative analysis of LRP16-GFP subcellular localization

MCF-7 cells were grown in 35 mm culture dishes and cotransfected with LRP16-GFP and K18 or pcDNA3 empty vector. 24 h after transfection cells were fixed with 3% formaldehyde (15 min) and nuclei were counterstained with 4',6'-diamidino-2-phenylindole dihydrochloride (DAPI). Cells were visualized under an inverted fluorescence microscope (IX-71; Olympus) equipped with a digital camera. The proportion of cells displaying LRP16-GFP in the nucleus was determined by counting at least 500 cells from each plate. The means and SEM were calculated from 3 separate plates from 3 independent experiments.

### Luciferase assays

MCF-7 cells were cultured in phenol-red free media stripped of steroids for at least 3 days and were then seeded into 35 mm culture dishes. Cells at 50% confluence were cotransfected by use of Superfect (Qiagen, Valencia, CA, USA). Cells were cotransfected with 0.5 μg of the reporter construct and 0.25 μg of ERα- and/or 0.5 μg of K18- or LRP16-expression vectors. Cotransfaction with plasmid pRL-SV40 (1 ng/per well) was used to control for transfection efficiency. Total DNA was adjusted to 2 μg per well with pcDNA3.1 empty vector. 36 h after transfection cells were treated with or without E2 (100 nM), cultured for a further 6 h, and cell extracts were prepared and relative luciferase activities were measured as described previously [19]. For knockdown experiments, 1 μg of siRNA duplexes, 0.5 μg of the reporter construct, 0.25 μg of the ERα-expression construct and 1 ng of pRL-SV40 were cotransfected using Lipofectamine 2000 according to the manufacturer's recommendations (Invitrogen). The total amount of nucleotides was adjusted to 4 μg per well with pcDNA3.1 empty vector. 42 h after transfection cells were treated with or without E2 (100 nM) and cultured for a further 6 h, harvested, and the relative luciferase activity was measured as described previously [19].

### Quantitative RT-PCR (qPCR)

Total RNA was extracted with use of TRIzol reagent (Invitrogen) and qPCR analysis was performed as described previously [15]. cDNA was prepared by use of Superscript II RNase H^- ^reverse transcriptase (Invitrogen) and 1-2 μg total RNA. The optical density was measured and equal amounts of cDNA were used in a normalization reaction with primers for *HPRT*. Oligonucleotide primers were as follows: *HPRT *sense, 5'-TTGCTCGAGATGTGATGAAAGGA-3'; *HPRT *antisense, 5'-TTCCAGTTAAAGTTGAGAGATCA-3'; *pS2 *sense, 5'-ATGGCCACCATGGAGAACAA-3'; *pS2 *antisense, 5'-TAAAACAGTGGCTCCTGGCG-3'; *cyclinD1 *sense, 5'-CTGGCCATGAACTACCTGGA-3'; *cyclinD1 *antisense, 5'-GTCACACTTGATCACTCTGG-3'; *c-Myc *sense, 5'-GACTATCCTGCTGCCAAGAG; and *c-Myc *antisense, 5'-TCGCCTCTTGACATTCTCCT-3'. Reactions were run on a LightCycler (Roche, Indianapolis, IN, USA). Experiments were performed in triplicate and repeated at least 3 times.

### Chromatin immunoprecipitation (ChIP) assays

MCF-7 cells (1 × 10^6^) were grown in 10 cm tissue culture plates in phenol-red free DMEM supplemented with 10% (v/v) steroid-depleted FBS. After 24 h the cells were transfected with 10 μg of pcDNA3.1-K18 or empty vector DNA using the Superfect reagent. 40 h later, transfected cells were treated with E2 (100 nM) for 1 h and were then analyzed by ChIP. Briefly, immunoprecipitation was carried out overnight at 4°C with ERα (Santa Cruz Biotechnology) antibody or nonspecific IgG antibody. DNA fragments were purified with use of a QIAquick Spin Kit (Qiagen). The presence of target gene promoter sequences in both input and recovered DNA immunocomplexes was detected by PCR. The promoter region (nt -353 to -30) of the *pS2 *gene was amplified.

### G1/S checkpoint assay

MCF-7 cells were cultured in phenol-red free medium stripped of steroids for at least 3 days, and were then seeded in 35 mm culture dishes and cotransfected with plasmids pcDNA3.1, pcDNA3.1-K18 and/or pcDNA3.1-LRP16 or K18-siRNA/control-siRNA. A vector expressing enhanced green fluorescent protein (EGFP) was used to identify transfected cells as described previously [49]. After 36 h, cells were treated with or without E2 (100 nM) for a further 12 and were then labeled with 10 μM BrdU for 2 h. Immunostaining was performed using anti-BrdU antibody (Becton Dickinson, Franklin Lakes, NJ, USA). The ratios of BrdU and EGFP double-positive cells to EGFP-positive cells were determined using an Olympus fluorescence microscope. At least 350 cells from each plate were counted. The means and SEM were calculated from 3 separate plates from 3 independent experiments.

### Statistical analysis

Results were expressed as the means ± standard error of the mean (SEM). Statistical analysis involved use of Statview 5.0 software. *P *< 0.05 was considered statistically significant.

## Authors' contributions

WH designed the study, performed the experiments, interpreted the data, and wrote the manuscript. YM, ZW and XY carried out some of the experiments and participated in data analyses and interpretation. YZ, MC, YS and JY performed some of the experiments. XF participated in data analysis and discussion and critically revised the manuscript. All authors read and approved the final manuscript.
